# CRISPR Technology in Gene-Editing-Based Detection and Treatment of SARS-CoV-2

**DOI:** 10.3389/fmolb.2021.772788

**Published:** 2022-01-11

**Authors:** Behrouz Shademan, Alireza Nourazarian, Saba Hajazimian, Alireza Isazadeh, Cigir Biray Avci, Mahin Ahangar Oskouee

**Affiliations:** ^1^ Department of Medical Biology, Faculty of Medicine, Ege University, Izmir, Turkey; ^2^ Department of Basic Medical Sciences, Khoy University of Medical Sciences, Khoy, Iran; ^3^ Immunology Research Center, Tabriz University of Medical Sciences, Tabriz, Iran; ^4^ Department of Microbiology, Faculty of Medicine, Tabriz University of Medical Sciences, Tabriz, Iran

**Keywords:** coronaviruses, SARS-CoV-2, CRISPR/Cas9, gene editing, ACE-2 receptors

## Abstract

Outbreak and rapid spread of coronavirus disease (COVID-19) caused by coronavirus acute respiratory syndrome (SARS-CoV-2) caused severe acute respiratory syndrome (SARS-CoV-2) that started in Wuhan, and has become a global problem because of the high rate of human-to-human transmission and severe respiratory infections. Because of high prevalence of SARS-CoV-2, which threatens many people worldwide, rapid diagnosis and simple treatment are needed. Genome editing is a nucleic acid-based approach to altering the genome by artificially changes in genetic information and induce irreversible changes in the function of target gene. Clustered, regularly interspaced short palindromic repeats (CRISPR/Cas) could be a practical and straightforward approach to this disease. CRISPR/Cas system contains Cas protein, which is controlled by a small RNA molecule to create a double-stranded DNA gap. Evidence suggested that CRISPR/Cas was also usable for diagnosis and treatment of SARS-CoV-2 infection. In this review study, we discoursed on application of CRISPR technology in detection and treatment of SARS-CoV-2 infection. Another aspect of this study was to introduce potential future problems in use of CRISPR/Cas technology.

## Introduction

Coronavirus disease (COVID-19) was spread in December 2019 and was recognized as a zoonotic disease (Drosten et al., 2017; Andersen et al., 2020). Severe acute respiratory syndrome (SARS) virus was detected in sputum samples in 2003, and advanced stages in fecal samples may have been transmitted to humans by an intermediate host such as bats and civets (Wang and Eaton, 2007; Graham and Baric, 2010). Severe acute respiratory syndrome coronavirus-2 (SARS-CoV-2) can be transmitted from an unknown carrier to a healthy person who could infect many people. SARS-CoV-2 resulted in pneumonia in Wuhan, China, with various symptoms reported. The disease has developed into a pandemic (Wu C. et al., 2020; Wu D. et al., 2020; Guan et al., 2020). Appropriate methods could treat and control the disease. CRISPR/Cas9 was first recognized as a microbial immune system through which these organisms acquire immunity to invading viruses and plasmids (Garneau et al., 2010). When the invaded foreign DNA enters the bacteria, it is cleaved by cas nuclease enzymes. A portion of the cleaved DNA is then placed between two repeating sequences at the CRISPR site. Here, it is called a spacer (Barrangou and Horvath, 2017; Shmakov et al., 2017). The spacer sequences are used as templates to generate short RNA sequences. These sequences direct the Cas protein to the invasive DNA. Once the Cas protein binds to the invasive DNA, the enzyme cuts the outer DNA sequence into both strands, creating the region of double-strand breaks (DSB) (Jinek et al., 2012; Wei et al., 2015; Brinkman et al., 2018) and the nucleotides at the DSB position change the structure or end codon in the gene. Non-homologous end-joining repair (NHEJ) or homology-directed repair (HDR) systems are induced to edit the genome to cut and cleave external DNA. These deletions and additions lead to a permanent change in the open reading frame (Hsu et al., 2014; Klein et al., 2019).

Some model systems, including mammalian cells, can efficiently cleave any complementary sequence to the gRNA and target and cleave the RNA. Targeted genome editing, often called CRISPR/Cas9, is increasingly recognized as an effective tool in medicine (Bawage et al., 2018; Freije et al., 2019). It can inactivate the SARS-CoV2 virus in mammalian cells by truncating the specific sequence of the virus. Cas proteins appear to help in detection and treatment of viral infections. They are introduced into the viral genome via guide RNAs and destroy it in the target regions (Aman et al., 2018; Xiao et al., 2018; Dolan et al., 2019). However, more regular interaction and collaboration between virology and molecular biology is needed to achieve substantial results in the treatment of SARS-CoV-2 (Rodrí guez-Rodr í guez et al., 2019). In this review, we first introduce general concept of CRISPR/Cas9. Then, we discuss potential challenges for treatment, and finally, we address prospects for CRISPR/Cas9-based antiviral strategies for SARS-CoV-2.

**FIGURE 1:**
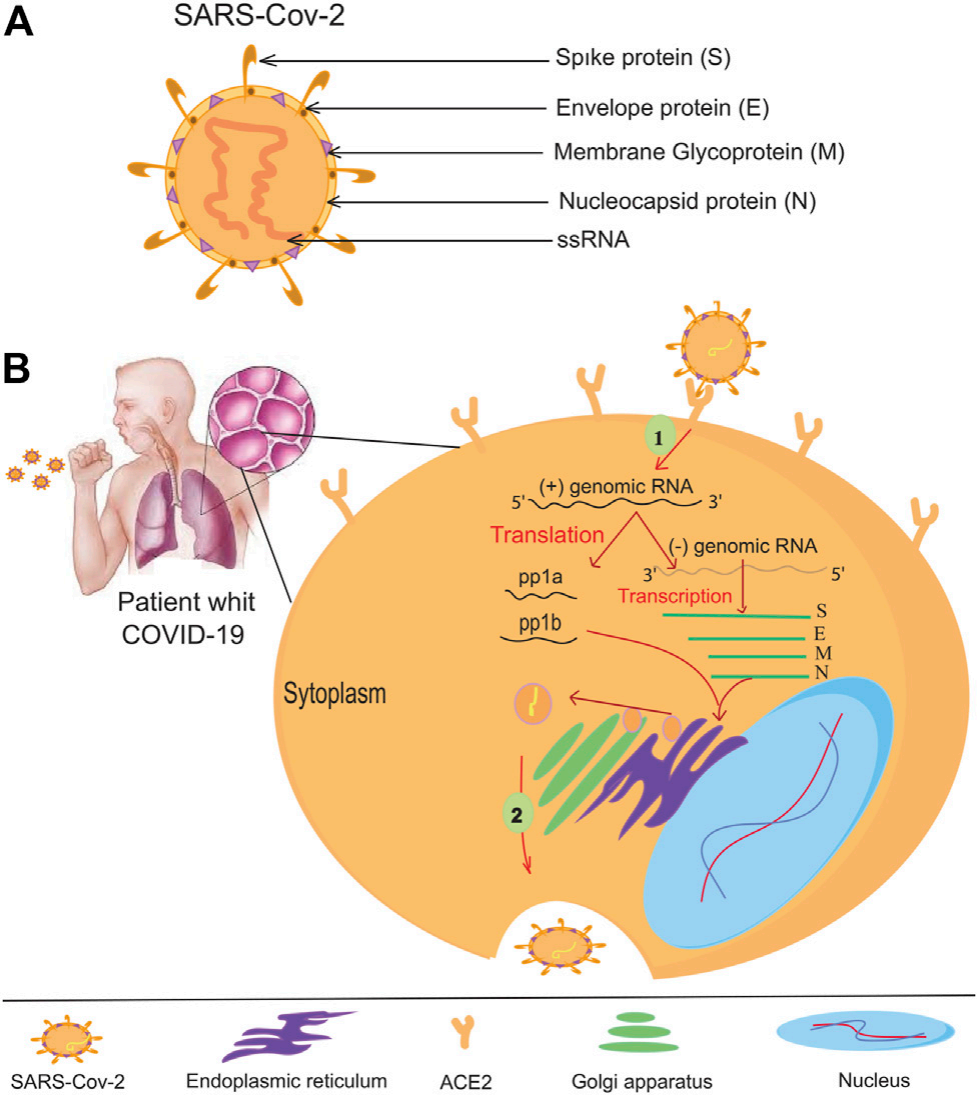
The coronavirus virion and its life cycle. **(A)** Typical structure and proteins of the coronavirus virion. The coronavirus genome encodes an (S) spike glycoprotein, an (E) envelope glycoprotein, an (M) membrane glycoprotein, and an (N) nucleocapsid protein. **(B)** To enter the host cells, the SARS-CoV-2 virus binds to the ACE -2 receptors of the host cell helped by protein (S), then the RNA of the virus enters the host cell. All viral products are provided at the expense of the host cell, so many viruses are produced by virus-infected cells.

The coronavirus family (CoVs) contains many virus species, and these viruses can cause various diseases in birds, livestock, and humans (Miłek and Blicharz-Domanska, 2018). Members of the family are spherical and have an approximate diameter of 125 nm. The spines protrude from the surface of the virion and give the virus a particular crown-like appearance (Neuman et al., 2006). There are four main structural proteins in coronavirus: Membrane (M), spikes (S), envelope (E), and nucleocapsid (N) proteins (Figure 1A). The spikes bind the virus to the host cell receptor (Beniac et al., 2006; Wrapp et al., 2020). Researchers have discovered human coronavirus receptors, such as angiotensin-converting enzyme 2 (ACE2) for SARS-CoV (Li et al., 2005) and HCoV-NL63 (Wu et al., 2009) or aminopeptidase N (APN) for HCoV-229E (Yeager et al., 1992; Li et al., 2005). ACE2, APN, and DDP4 are ectopeptidase enzymes with different functions expressed on the surface of various cell types, including those of the human respiratory tract. MERS-CoV has been shown to require a dipeptidyl peptidase-4 (DPP-4) surface receptor to enter the host cell. SARS-CoV-2 uses the endogenous enzyme angiotensin-converting enzyme 2 (ACE2) to enter host cells (Raj et al., 2013; Monteil et al., 2020). After binding to the appropriate receptor, a fusion of the virus and cell membrane occurs, and the viral genome is transferred to the host cell’s cytoplasm. In the host cell, the viral products are produced and assembled (Belouzard et al., 2009). After the virus particles are assembled, they are transported to the cell surface by vesicles and released by exocytosis (Fehr and Perlman, 2015). SARS-CoV-2 mainly infects epithelial cells in the lung but can also invade macrophages and dendritic cells (Weinheimer et al., 2012; Zhou et al., 2015). The exact mechanism of lung injury caused by SARS-Cov-2 is still unknown (Zhou et al., 2020). Serologic evidence of SARS-CoV-2 infection was observed in some individuals at animal markets before the disease outbreak. Some animals have also been infected with SARS-CoV-2 viruses isolated from camels, Himalayan palm civets, and raccoon dogs (Azhar et al., 2014; Fung et al., 2020). Further evidence from phylogenetic analysis suggests that SARS-CoV-2 is bat-derived (Lau et al., 2005; Zheng, 2020), as the genetic similarity between SARS-CoV-2 and bat-SARS is greater than 95%. Bat-SARS has the potential to be transmitted to humans. Although the risk of transmission is lower, it is affected by bat SARS in an area (Cook et al., 2021). Although one study has shown that SARS-CoV-2 is not a mosaic (Paraskevis et al., 2020), there is speculation about its heritability. The UK Medicines Agency has approved molnupiravir to treat mild to moderate COVID-19 in people with at least one risk factor for severe disease. Molnupiravir could cut the number of people who need to go to the hospital in half and reduce the number of deaths. However, the supply would not last long if it were given to everyone who is sick because the daily caseload is high. Use of the drug would likely be limited to those at the highest risk for disease complications, such as older adults with heart, lung, or kidney disease, diabetes, or cancer (Wu F. et al., 2020; Mahase, 2021). Vaccination is the most important method of epidemic control. The emergence of numerous SARS-CoV-2 variants that are less prone to disease- and vaccine-induced immunity threatens progress. Despite these ongoing threats, the efficacy of the SARS-CoV-2 vaccine provides a reason for optimism for 2021 (Creech et al., 2021). SARS-CoV-2 neutralizing antibodies in the serum of cured patients can be recovered and reused if SARS-CoV-2 recurs (Zhou and Zhao, 2020). Such antibodies will help protect individuals at high risk.

## Application of CRISPR/Cas9 Technology in Current Virology

Host-virus conflict is a dynamic process. Viruses use host factors to complete their life cycle, and the host uses the body’s immune system to fight off the viral infection, so it does not waste energy. The virus must enter the host cell to replicate its genome and complete its life cycle (Sicard et al., 2019) (Figure 1B). Theoretically, antiviral treatments can prevent the virus from entering the host cell or destroy the genetic elements of the virus. Targeting host factors can help the virus become resistant to antiviral drugs (Lin and Gallay, 2013). However, this hypothesis needs further investigation to identify its weaknesses and exploit them after these weaknesses have been addressed. The CRISPR/Cas9 system could be useful because it targets viral nucleic acid and host material quickly and conveniently (Lino et al., 2018). Cas9 is known in CRISPR/Cas systems as a DNA endonuclease directed from a guide RNA (sgRNA) to the target DNA to alter the genome of the target region (Kennedy and Cullen, 2015; Ishino et al., 2018). This genome editing leads to an antiviral status within the host cell. CRISPR/Cas9 was the first system studied in HIV-1 gene therapy research (Xiao et al., 2019). Host cell receptors, CCR5 and CXCR4, help HIV enter the host cell (Wilen et al., 2012; Santos-Costa et al., 2014). Therefore, one of the antiviral candidates to treat HIV is suppressing these receptors. Researchers have successfully suppressed the expression of CCR5 in primary CD4 T cells at an appropriate level using the CRISPR/Cas9 gene-editing system. These cells develop resistance to HIV-1 and do not cause extracellular toxicity (Hou et al., 2015; Li et al., 2015).

Host cell factors such as the Apo-Lipoprotein B Editing Complex (APOBEC3) and Tripartite Motif Containing 5 (TRIM5) have been identified as viral limiters for HIV infection (Bogerd et al., 2015). APOBEC3 is thought to act as an antiviral agent by causing mutations in the viral genome. CRISPR/Cas9-based regulation of the host APOBEC3 factor reduces HIV reporter gene expression and provides antiviral effects (Jern et al., 2009; Bogerd et al., 2015). Two specific amino acids in TRIM5 have made it an actual antiviral agent against HIV-1 infection. This antiviral candidate can induce cleavage of viral capsid proteins, demonstrating its antiviral properties (Sastri and Campbell, 2011; Weatherley et al., 2017). Therefore, TRIM5 may be a suitable target for the CRISPR/Cas9 system. Eliminating microRNA-146 by CRISPR/Cas9 resulted in a significant increase in HIV-1 limiting factors (Teng et al., 2019).

Some host factors are essential for virus replication, assembly, and budding. Therefore, knocking out their genes could be an alternative to preventing HIV-1 infection (Lin and Nagy, 2013; Xu and Nagy, 2015); such gene deletions can be performed using the CRISPR/Cas9 system (Gilani et al., 2019). Interestingly, these gene deletions have no significant effects on host cells (Chen J. S. et al., 2018). These results demonstrate the antiviral potential of CRISPR/Cas9-based therapies, as it is possible to disrupt key host factors essential for HIV infection.

## Application of CRISPR/Cas Technology in SARS-CoV-2

The worldwide pandemic of SARS and CoV-2 poses a significant threat to global public health and societal stability and has become a significant global public health problem. Regrettably, current diagnostic and therapeutic methods to prevent and control SARS-CoV-2 have many limitations. CRISPR/Cas technology has emerged as a potential complement to conventional methods in recent years. Biomedicine has extensively used biological tools based on the CRISPR/Cas systems. They are helpful in pathogen detection, clinical antiviral treatment, and drug and vaccine discovery. Therefore, CRISPR/Cas technology could be promising in preventing and treating SARS-CoV-2 and other emerging infectious diseases.

## Application of CRISPR in the Detection of SARS-CoV-2

**FIGURE 2:**
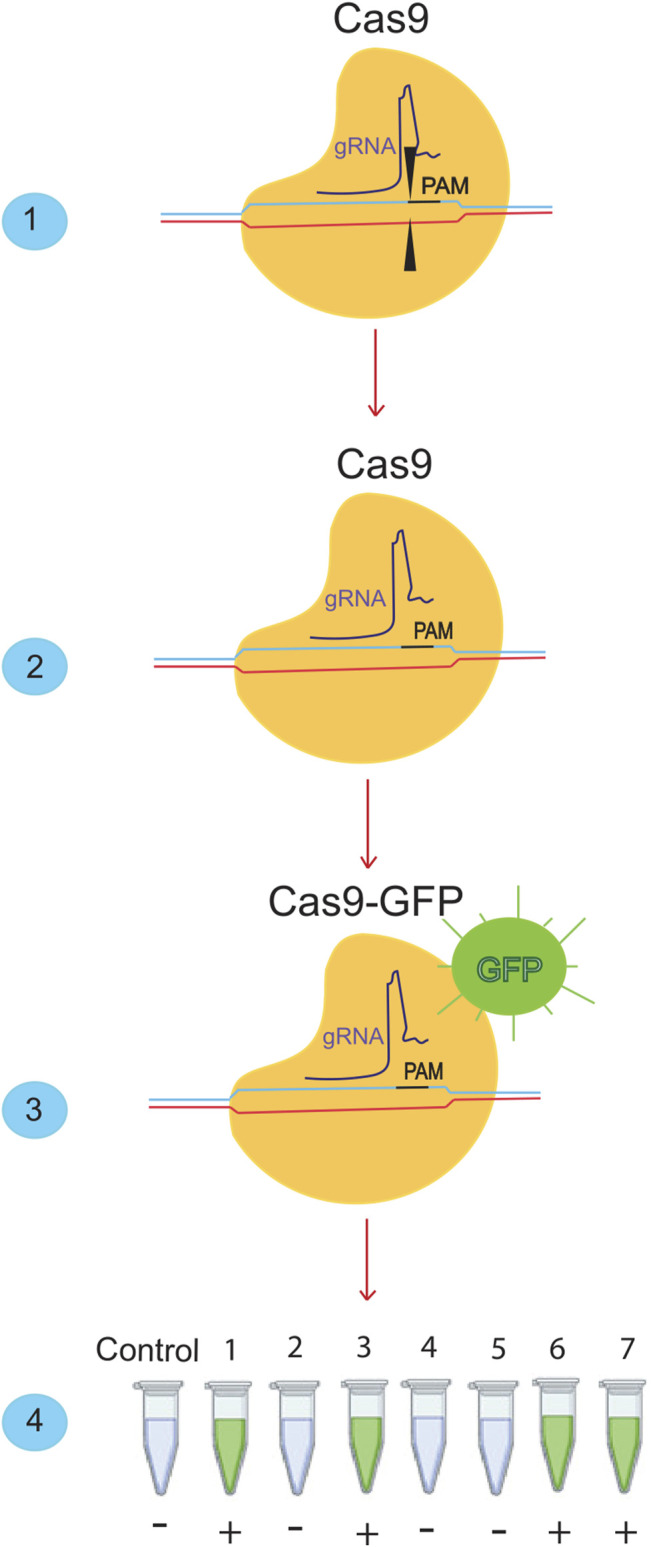
Detection of SARS-cov-2 using the Crisper/Cas system. (1) The Crisper/Cas9 system binds to specific sequences using gRNA. (2) If we remove the cutting effect from this system and (3) add a fluorescent dye, we can bind it to the desired sequences. When it is bound to the desired sequence, it will produce a green color. (4) With this ability of the CRISPR/CAS9 system, many SARS-CoV-2 samples can be detected in a short time.

CRISPR-Cas systems could be used for molecular diagnosis of nucleic acids (Figure 2) (Jia et al., 2020:; Kaminski et al., 2021). CRISPR-Cas-based diagnostic methods have the same sensitivity and specificity as conventional PCR. However, their cost is low because they do not require complex or expensive technology (Ayanoğlu et al., 2020). The application of CRISPR-Cas in molecular diagnostics could transform global diagnostic and healthcare systems (Gootenberg et al., 2017). The Cas proteins used in CRISPR-Cas systems vary depending on the DNA or RNA targeted and the intended applications (Makarova et al., 2015). Following the global spread of the COVID-19 pandemic, rapid and straightforward diagnostic techniques are in high demand. CRISPR-based methods, which have demonstrated superior detection capability in as little as 30–60 min, could overcome this obstacle. In addition, a CRISPR/Cas9-mediated lateral flow nucleic acid assay (CASLFA) has been developed to identify infections using the CRISPR/Cas system (Wang et al., 2020). However, FDA approval is still pending. Regarding “collateral breast activity,” CRISPR-based diagnostic techniques have been developed using the Cas12a or Cas13 nuclease. The Cas12a/Cas13 nuclease, a component of the CRISPR tool, is activated after CRISPR RNA binds to the target bosome (crRNA). When produced, it non-specifically cleaves ssDNA/RNA particles in the vicinity and explicitly acts as a collateral bosome or transbosome. Researchers took advantage of this property to develop fluorescently labeled ssDNA/RNA press reporter probes capable of detecting visible bands in a paper strip via a side-stream assay, enabling the development of a novel nucleic acid-based diagnostic test (Chen S. et al., 2018). Viral RNA targeting crRNA can activate Cas protein, resulting in collateral cleavage of press reporter probes and a helpful band on the paper strip (Chen J. S. et al., 2018). In the newly developed Specific High-sensitivity Enzymatic Reporter Unlocking (SHERLOCK) technology, the activity of the crRNA-Cas13a protein complex is used to recognize RNA molecules and cut collateral RNA near the target RNAs. Metsky et al. have developed a website with CRISPR-Cas13-based assay designs for the detection of 67 diseases, including SARS-CoV-2, Zika virus, and dengue fever, with a choice of single or multiple panels (Metsky et al., 2020). The comprehensive SARS-CoV-2 diagnostic test is based on advanced technology from SHERLOCK. This technique uses fluorescently identified, non-targeted press reporter RNA (Kellner et al., 2019). SHERLOCK test for detecting SARS-CoV-2, which has a sensitivity of 10 copies per microliter and can be fluorescently confirmed, has been validated with counterfeit RNA fragments. This molecular analytical test should be inexpensive and provide rapid results. The DETECTOR is a similar technique used to amplify pathogenic DNA with RPA, and reverse transcription to identify RNA viruses is also used as a SHERLOCK system in this procedure. Cas12a-crRNA identifies the target and activates the Cas12a nuclease, which cleaves fluorescently labeled reporter sDNA without discrimination. In less than an hour, DETECTOR distinguished between human papillomavirus 16 (HPV16) and human papillomavirus 18 (HPV18) in pure DNA from cultured human cells and professional samples (Myhrvold et al., 2018). Broughton et al. diagnosed COVID -19 using two specific crRNAs targeting genes E and N and a discovery series ranging from 70 to 300 copies per microliter of sample material. They used LAMP with reverse transcription instead of RPA-based amplification to identify COVID -19 in less than 30 min. These CRISPR-Cas-based nucleic acid detection methods require independent amplification of nucleic acids. They require human activity, complicating detection and increasing the risk of spreading contamination (Broughton et al., 2020a). Ding et al. developed the AIOD-CRISPR (All-In-One Dual CRISPR-Cas12a) assay method, which enables rapid visual detection of viral nucleic acids with high sensitivity and accuracy. In this article, all materials required for viral nucleic acid detection are incubated in a single pot at 37°C, which simplifies the process and minimizes the risk of contamination. With high sensitivity, SARS-CoV-2 genomic RNA was detected with the AIOD-CRISPR assay (Ding et al., 2020). LED assay uses blue light illumination to image the tubes instead of a paper dipstick with sidestream detection. Scientists in India have identified the Francisella novicida Cas9 orthology (FnCas9) as sensitive to nuclear differences, and the Linked Attire Discovery Assay (FELUDA) for FnCas9 has been developed as a low-cost point-of-care (LCC) assay for identifying SARS-CoV-2 infections in the clinical setting (Azhar et al., 2020).

## CRISPR’s Advantages in Detection of SARS-CoV-2

“Metagenomics” and “qRT-PCR” are two widely used molecular methods for identifying novel viruses (Gu et al., 2019; Corman et al., 2020). Current qRT-PCR-based SARS-CoV-2 diagnostic methods are efficient and accurate for virus detection. Where qRT-PCR technology is not available, the virus can spread globally (Lucia et al., 2020). Finally, the diagnostic accuracy of many molecular methods has not yet been clarified. Although CRISPR/Cas has been a widely used gene-editing strategy since 2013, the simultaneous promiscuous cleavage tasks of a specific collection of Cas nucleases were discovered later and used to detect nucleic acids from artificial insemination (Chen S. et al., 2018; Harrington et al., 2018). Due to its exceptional sensitivity, specificity, and reliability, RNA-directed nucleic acid detection based on CRISPR/Cas nuclease has recently shown significant potential for developing next-generation molecular diagnostic technology (Ding et al., 2020; Lucia et al., 2020). For example, AIOD-CRISPR can detect only 1.2 copies of DNA targets and 4.6 documents of RNA targets in 40 min of incubation without preamplification when detecting SARS-CoV-2 (Lucia et al., 2020). CRISPR-nCoV has the same sensitivity and uniqueness as next-generation metagenomic sequencing (mNGS) in less than 40 min (Hou et al., 2020). Some of the major advantages of this approach over existing techniques such as qRT-PCR are 1) uniformity of signal in a nucleus (e.g., SARS-CoV-2 guide RNAs can be distinguished from SARS-CoV and MERS-CoV at the N_2_ site) and 2) integration with low-cost portable reporting sheets and side streptometers. 3) isothermal signal amplification for rapid target detection in the absence of termocycling. CRISPR-based assays are more accessible and convenient than RT-PCR viral RNA identification assays because the CRISPR system does not require bulky instrumentation or complicated processes (Ganbaatar and Liu, 2021). Most CRISPR-Cas-based detection methods require pre-amplifying a specific nucleic acid combination and manual procedures. These modifications will undoubtedly complicate procedures and impose costs on the environment. The following table summarizes the current studies on the diagnosis of SARS-CoV-2.

### Sensitive and Specificity

CRISPR-based diagnostic assays show excellent clinical sensitivity and specificity (Huang W. et al., 2020; Joung et al., 2020a; Patchsung et al., 2020). To improve the sensitivity and specificity of CRISPR-based SARS-CoV-2 detection, different models were created. Selection of two crRNAs improved sensitivity (Huang Z. et al., 2020) and increased resistance to viral RNA changes (Ooi et al., 2020).

In addition, several improvements have been made to increase the sensitivity of CRISPR-based SARS-CoV-2 detection experiments. These include modifying crRNA (Nguyen et al., 2020), incorporating small particles to improve action kinetics (Joung et al., 2020b), improving reagent ratios (Huang W. et al., 2020), increasing reagent concentration through careful focusing (Ramachandran et al., 2020), and increasing RNA input besides RNA quantity. Computational techniques ensure the sensitivity and specificity of amplification primers and crRNAs to detect SARS-CoV-2 (Ackerman et al., 2020; Arizti-Sanz et al., 2020; Metsky et al., 2020). Researchers developed customized CRISPR-based assays with sensitivity and specificity comparable to quantitative real-time PCR (qPCR) based on these findings. Incorporating the methods described above could help improve the overall sensitivity and specificity of COVID -19 CRISPR diagnostic tools.

### Turn-Around Time

Several CRISPR-mediated COVID -19 diagnostic studies have used the same techniques as RT -qPCR to recover viral RNA, consistent with previous findings (Ali et al., 2020; Huang Z. et al., 2020). Unlike rapid RNA extraction techniques needed for point-of-care diagnostics, these methods are time-consuming. Therefore, several researchers have investigated whether CRISPR-based assays are feasible using rapid viral RNA extraction methods. When Joung and colleagues mixed the clinical samples with the Quick Extract solution, they incubated them at 95°C for 5 min before assaying them for viral RNA. Heat treatment and chemical reduction were performed as a 10-minute technique lysed the viral particles and inactivated the nucleases (Arizti-Sanz et al., 2020). In one study, Ramachandran et al. used electric field-driven microfluidics to recover viral RNA in less than 5 min (Ramachandran et al., 2020). RT-qPCR to detect SARS-CoV-2 takes approximately 45 min if RNA extraction is omitted (Corman et al., 2020). This result, considering that RT-qPCR is compatible with rapid RNA extraction methods, suggests that the time required for RNA extraction in CRISPR-based research equals that of RT-qPCR (Ladha et al., 2020). The time required for an essay varies depending on the subject. However, specific efficient procedures can be completed in less than 30 min (Broughton et al., 2020b). Others require 40, 45, or 50–60 min without RNA extraction (Arizti-Sanz et al., 2020; Patchsung et al., 2020). One study found that an automated CRISPR-based assay can be performed in 30 min (Ramachandran et al., 2020). CRISPR-based SARS-CoV-2 detection methods equal RT-qPCR in terms of assay time. RT-qPCR analyses require sending samples to a central laboratory. However, CRISPR-based diagnostics allow on-site detection, which drastically reduces reporting time.

### Ease of Use

RT-qPCR experiments are performed as one-step reactions using master mixes to simplify them. A master mix containing both RT-LAMP and Cas12a-based detection reagents was developed by Joung and colleagues and proved stable after six freeze-thaw cycles (Joung et al., 2020a). Most CRISPR-based SARS-CoV-2 detection assays require two phases, but researchers have also developed one-step methods that require less time and effort (Arizti-Sanz et al., 2020; Joung et al., 2020b). A wide range of CRISPR-based assays can be performed using a single method, with RT-qPCR requiring less time or the same time as RT-PCR due to preparing the required reagents in master mixes. CRISPR-based assays are comparable to RT-qPCR in terms of ease of use. However, point-of-care assays require fewer manual activities and a lower level of technical skill. CRISPR-based assays are being developed that are both automated and sample-to-result (Ramachandran et al., 2020).

### Requirement of Equipment

Since most CRISPR-based research uses isothermal techniques, a thermocycler is not required. So, it’s possible to use a normal heating block or water bath to perform the tests (Ali et al., 2020; Metsky et al., 2020). When using a lateral flow readout technique, no signal detection equipment is required. After DNA amplification, the reaction tube must be opened, which can lead to contamination and false-positive results in subsequent tests. Therefore, reading lateral flow strips requires a specific position or a closed cartridge. A fluorescence readout would be more appropriate. Although a plate reader primarily detects the fluorescent signal, many studies have shown that it can also be identified by eye examination under blue light (Ding et al., 2020; Wang et al., 2020).

Viral RNA can be isolated from clinical samples using techniques that do not require complex or lengthy equipment for the CRISPR-based identification of SARS-CoV-2. Compared with traditional, labor-intensive RNA extraction techniques, these rapid extraction methods have the same (Joung et al., 2020b) or slightly lower efficiency (Guo et al., 2020).

Microfluidic devices that extract viral RNA and enable CRISPR-based detection can be used for automated or sample-to-result assays (Ramachandran et al., 2020). Researchers used a battery-powered, portable thermal cycler and fluorescence reader (Rauch et al., 2020). Finally, these examples demonstrate that tests to detect SARS-CoV-2 with CRISPR do not require expensive or complicated equipment. These tests can be performed in locations other than a central laboratory, such as airports, clinics, and other locations with limited resources.

### Cost per Test

Although using lateral flow strips increases the cost per assay (Ooi et al., 2020), the total material cost for fluorescence-based CRISPR-mediated SARS-CoV-2 detection assays is lower than the material cost for RT -qPCR-based SARS-CoV-2 detection assays (Hou et al., 2020; Ooi et al., 2020). For example, the CRISPR-COVID assay costs less than $3.50 for a single reaction, depending on the assay technique (Hou et al., 2020). On an industrial scale, the cost can be as low as $0.6 per pound (Gootenberg et al., 2020; Kellner et al., 2019). In addition, CRISPR-based screening has reduced the cost of the first tool (Guo et al., 2020). (Guo et al., 2020). Fluorescence-based CRISPR-mediated COVID–19 analysis assays are less costly than RT–qPCR.

## Application of CRISPR in SARS-CoV-2 Treatment

SARS-CoV-2 is a novel coronavirus of the positive-sense RNA virus family that infects the respiratory tract and causes disease through direct cytotoxic effects and the production of host cytokines (Liu et al., 2020). The life cycle of SARS-CoV-2 is like that of other strongly associated coronaviruses, such as the virus that causes SARS. The virus spreads its RNA genome in the cell, synthesizes the genomic and subgenomic negative sense RNAs used in the viral mRNA, and produces a new copy of the viral positive sense genome (Du et al., 2009; Mocarski et al., 2020). While conventional vaccines recognize viral proteins or viruses by activating the human immune system and limiting viral entry into the cell (Rappuoli, 2018), the CRISPR-based approach is an alternative antiviral strategy to recognize and eliminate the viral genome and mRNAs within the cell. It should be possible to restrict viral replication to specific positives and viral mRNAs while destroying viral genome replication and gene expression templates. Therapeutic applications of CRISPR are on the rise. Technology plays an essential role in exploring potential therapies for various genetic diseases through direct modification of the genome (Straiton, 2019).

**FIGURE 3:**
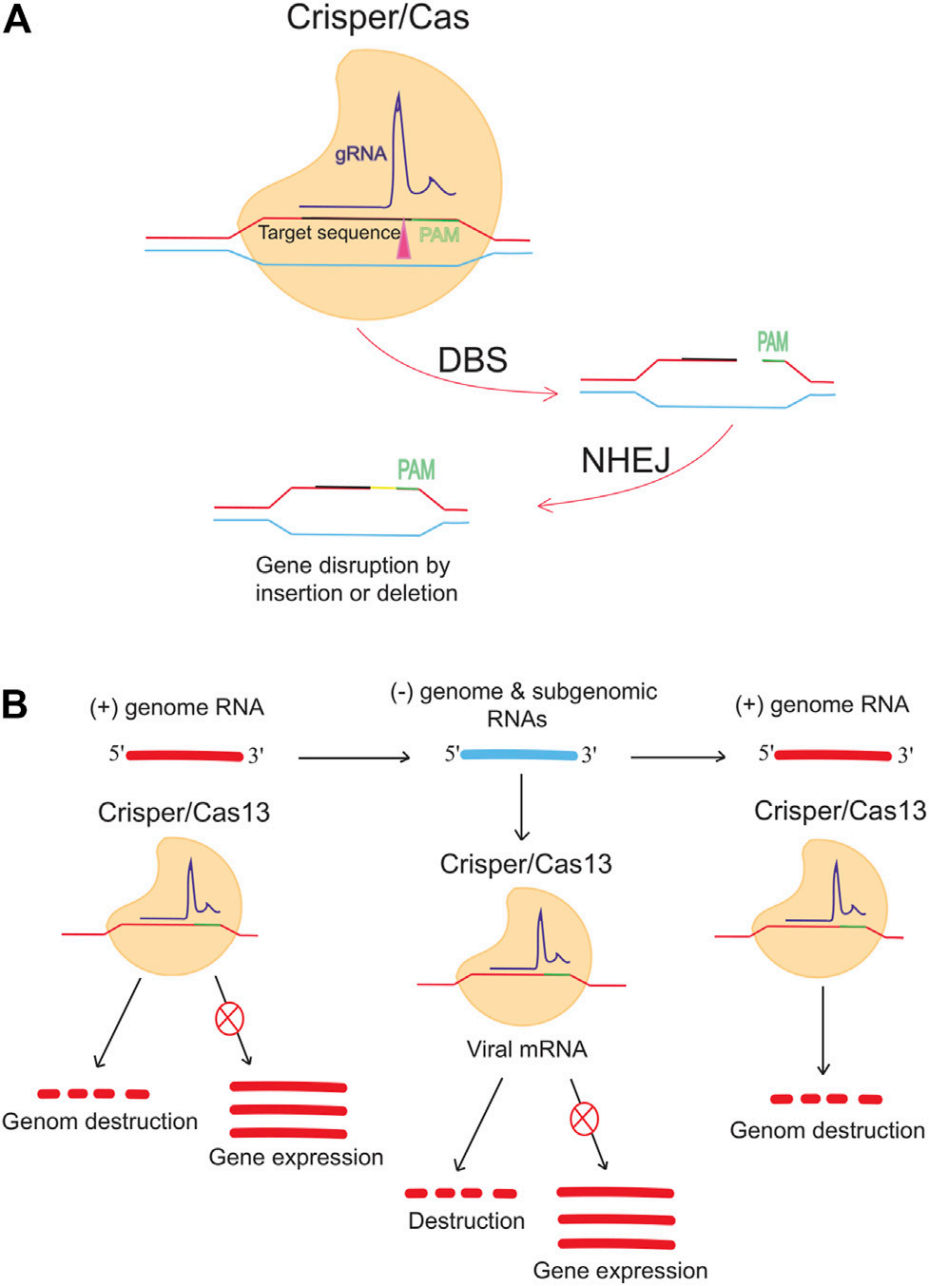
Anti-COVID-19 genome editing was performed using the Crisper/Cas system and PAC-MAC function. **(A)** A mutation in the target gene is caused by the CRISPR/CAS9 system, which is supported by the NHEJ repair mechanism and causes a change in the gene. The gene expression is reduced or stopped depending on the target. **(B)** Cas13d can inhibit viral activity and replication. Cas13d targets and cleaves 100% of the positive sense RNA produced by viruses.

Besides DNA targeting Cas9, RNA targeting CRISPR-Cas13 is an antiviral approach against single-stranded RNA viruses such as lymphocytic choriomeningitis virus (LCMV), influenza A virus (IAV), and vesicular stomatitis virus (VSV) in human cells (Freije et al., 2019). Conversely, Stanford College (CA, United States) researchers are working on CRISPR-based therapies for infectious diseases, using a different method and going beyond the human genome. When researchers worked on the flu virus, they followed in the footsteps of many others. They shifted the focus of their gene-targeted antiviral drug to COVID-19 and the pandemic (Abbott et al., 2020a; Abbott et al., 2020b). It was reported that the prophylactic CRISPR antiviral approach in human lung epithelial cells (PAC-MAN) was identified as a potentially helpful new technique to stop viral traits and replication and that the PAC-MAN approach was identified as a type of genetic intervention to target SARS-CoV-2 and potentially all sequenced coronaviruses (Figure 3). Interestingly, a pool of crRNA suppressed about 70% of the reporter signal, demonstrating the potential of CRISPR PAC–MAN technology to degrade viral genetic material. In addition, several crRNAs targeting the entire conserved region of the SARS-RdRP CoV-2 and N-protein genes caused RNA degradation of over 80 and 90%, respectively.

To reprogram CRISPR-Cas13b against the genomic and subgenomic RNAs of SARS-CoV-2, we performed genome-wide computational predictions and screens at single-nucleotide resolution. Cas13b effectors reprogrammed to target accessible segments of spike and nucleocapsid transcripts had silencing efficiencies greater than 98 percent in virus-free animals. Tailored and multiplexed Cas13b CRISPR RNAs (crRNAs) inhibit viral replication in mammalian cells infected with replication-competent SARS-CoV-2, including novel dominant variants. CRISPR-Cas13-based viral suppression strategy is readily adaptable and can be extended to harmful viruses other than SARS-CoV-2 and, therefore, could provide an effective platform for antiviral treatments (Fareh et al., 2021). However, it is critical to identify and study the deleterious effects of using single-guide RNAs (sgRNAs), the CRISPR/Cas system, or PAC-MAN on host physiology. Although the Cas method seems to have a high chance of successfully identifying therapies, it needs further investigation.

However, it is critical to identify and investigate the deleterious effects of using single-guide RNAs (sgRNAs), the CRISPR/Cas system, or PAC-MAN on host physiology. Although the Cas method seems to have a high chance of successfully identifying therapies, it needs further investigation.

## Limitation of CRISPR Technology

CRISPR technology is advancing rapidly. Although recently discovered and new, CRISPR/Cas is a tool with multiple genome engineering capabilities. Because of its ability to edit genomes in such a user-friendly way, it has attracted the attention of biomedical researchers. CRISPR can appropriately solve various viral diseases. In cell-based and animal studies, successful results have been achieved in several human viral infections (Scheufele et al., 2017; Brokowski, 2018; Li et al., 2019). The therapeutic use of CRISPR/Cas to treat human viral diseases has generally gained great importance (Scheufele et al., 2017). However, gaining expertise in the diagnostic and therapeutic use of CRISPR/Cas in viral infections is associated with potential risks. Because this is a new science, we will briefly describe the limitations of CRISPR/Cas. We hope these limitations will be addressed, and an appropriate therapeutic and diagnostic system for SARS-CoV-2 will be developed. There are several legitimate concerns about CRISPR technology’s efficacy and technical limitations. According to research, both target and off-target editing provide limited and partial results, and CRISPR studies in animals and human cells have demonstrated these limitations (Guo and Li, 2015; Peng et al., 2016; Bohaciakova et al., 2017; Zischewski et al., 2017).

Although few studies have shown off-target editing and most studies support CRISPR/Cas, one of the major concerns associated with the CRISPR/Cas system is the possibility of off-target activity and mutant viruses. Viral escape mutations are caused by deletions (indels) at the Cas9 segregation site (Guo and Li, 2015). Using NHEJ repair system, Cas9 causes a mutation and renders the virus ineffective. Typically, NHEJ repair system repairs the damage (Ingram et al., 2019). However, a subset of these mutations can cause the virus to survive and escape, and viruses with such mutations are no longer interested in the original gRNA (De Silva Feelixge et al., 2018). Thus, inappropriate mutations can occur with any virus. If these cells are infected with the mutant virus, they might resist CRISPR/Cas treatment. Therefore, the desired results may not be achieved. Non-mutated virus-infected cells may provide a viral reservoir for disease spread in subsequent disease episodes (Allocati et al., 2016). Therefore, CRISPR/Cas system is risky and not profitable in the antiviral market.

Transferring CRISPR/Cas9 to virus-infected cells is another limitation of this new method. The success of this technology in the clinical setting is necessary to control the most severe viral diseases, including SARS-CoV-2 (Rath et al., 2015). As CRISPR becomes more efficient and sensitive, these concerns may become obsolete. Technology is advancing at an unprecedented pace.

## Conclusion

Emerging viruses such as SARS-CoV-2 are responsible for hundreds of thousands of illnesses and deaths worldwide each year. The disease is spreading everywhere and destroying the economies of affected populations. Genome editing strategies to deactivate the viral genome could be a suitable way to treat such diseases. The lack of effective drugs and vaccines may be contributing to so many SARS-CoV-2 samples being collected for rapid diagnosis. Containing and preventing further spread of the virus appears to be critical, and rapid detection of infection in organisms may prevent further spread of the disease. CRISPR/Cas is a solution for treating virus-related diseases with many future applications. Therefore, the CRISPR/Cas9 system could be helpful, especially if some valuable and specific sgRNAs are developed. If the CRISPR/Cas system leads to therapeutic and diagnostic solutions, the financial burden will be reduced because CRISPR/Cas-based therapies will eliminate the need for drugs. In addition, the treated individual will not suffer repeated disease relapses. Therefore, advances in the CRISPR/Cas system and the success of clinical trials in animal models are critical Table 1, Guanghui et al., 2020.

**TABLE 1:** Some CRISPR-based SARS-CoV-2 diagnostic studies.

CRISPR/System	Sample type	Number of samples	Assay time	Platform	Specific/Sensitive	Country	References
CRISPR–Cas12a	respiratory swab	36	<40 min	DETECTR	—	United States	Broughton et al. (2020b)
CRISPR–Cas13a	nasopharyngeal swabs	154	>60 min	SHERLOCK	100%/96%	Thailand	Patchsung et al. (2020)
CRISPR–Cas13a	nasopharyngeal swabs	1808	110 min	CREST	100%/88.8%	United States	Rauch et al. (2020)
CRISPR–Cas13a	nasopharyngeal swabs	50	50 min	SHINE	100%/90%	United States	Arizti-Sanz et al. (2020)
CRISPR–Cas12b	nasopharyngeal or anterior nasal swab	202	<60 min	STOPCovid	98.5%/93.1%	—	Joung et al. (2020a)
CRISPR-Cas3 And CRISPR–Cas12a	nasopharyngeal and oropharyngeal swab	31	40 min	CONAN	95%/90%	Japan	Yoshimi et al. (2020)
CRISPR–Cas12a	nasopharyngeal swabs, sputum, BAL	378	30 min	DETECTR	95.5%/93%	Dutch	Brandsma et al. (2020)
CRISPR/Cas12a	Clinical sample	31	45 min	CRISPR/Cas12a-NER	100%/100%	China	Wang et al. (2020)
CRISPR/Cas12a	raw nasopharyngeal swab	8	35 min	ITP-CRISPR	100%/75%	United States	Ramachandran et al. (2020)
CRISPR/Cas12a	Pharyngeal swab, nasopharyngeal swabs	295	60 min	SENA	100%/100%	China	Huang et al. (2020a)
CRISPR–Cas13a	nasopharyngeal swab, bronchoalveolar lavage fluid specimens	114	40 min	CRISPR-COVID	100%/100%	China	Hou et al. (2020)

BAL, broncheo-alvealar lavage.
